# Can predators stabilize host–parasite interactions? Changes in aquatic predator identity alter amphibian responses and parasite abundance across life stages

**DOI:** 10.1002/ece3.9512

**Published:** 2022-11-15

**Authors:** Miranda Strasburg, Michelle D. Boone

**Affiliations:** ^1^ Department of Biology Miami University Oxford Ohio USA

**Keywords:** anurans, carryover effects, host–parasite interactions, predator–prey interactions, trematodes

## Abstract

The role of parasites can change depending on the food web community. Predators, for instance, can amplify or dilute parasite effects on their hosts. Likewise, exposure to parasites or predators at one life stage can have long‐term consequences on individual performance and survival, which can influence population and disease dynamics. To understand how predators affect amphibian parasite infections across life stages, we manipulated exposure of northern leopard frog (*Rana pipiens*) tadpoles to three predators (crayfish [*Orconectes rusticus*], bluegill [*Lepomis macrochirus*], or mosquitofish [*Gambusia affinis*]) and to trematode parasites (*Echinostoma* spp.) in mesocosms and followed juveniles in outdoor terrestrial enclosures through overwintering. Parasites and predators both had strong impacts on metamorphosis with bluegill and parasites individually reducing metamorph survival. However, when fish were present, the negative effects of parasites on survival was not apparent, likely because fish altered community composition via increased algal food resources. Bluegill also reduced snail abundance, which could explain reduced abundance of parasites in surviving metamorphs. Bluegill and parasite exposure increased mass at metamorphosis, which increased metamorph jumping, swimming, and feeding performance, suggesting that larger frogs would experience better terrestrial survival. Effects on size at metamorphosis persisted in the terrestrial environment but did not influence overwintering survival. Based on our results, we constructed stage‐structured population models to evaluate the lethal and sublethal effects of bluegill and parasites on population dynamics. Our models suggested that positive effects of bluegill and parasites on body size may have greater effects on population growth than the direct effects of mortality. This study illustrates how predators can alter the outcome of parasitic infections and highlights the need for long‐term experiments that investigate how changes in host–parasite systems alter population dynamics. We show that some predators reduce parasite effects and have indirect positive effects on surviving individuals potentially increasing host population persistence.

## Graphical abstract

Using an amphibian‐trematode model system, our study demonstrates that predators can alter the outcome of parasitic infections and highlights the need for long‐term experiments that investigate how changes in host‐parasite systems alter population dynamics. Predator identity matters; some predators reduce parasite effects and have indirect positive effects on surviving individuals increasing host population persistence.

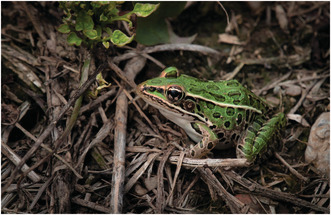

## 1 INTRODUCTION

Hosts and parasites coexist in complex ecological communities that can amplify or dilute parasite abundance within hosts directly through impacts on host and parasite responses or indirectly through food web effects (Civitello et al., 2015; Johnson et al., 2015; Keesing et al., 2010). Predicting infection risk is particularly complicated in systems that require multiple species for infection to occur. Because of unprecedented global changes in biodiversity (Dornelas et al., 2014) and increases in disease and parasite outbreaks (Jones et al., 2008), understanding how community‐level changes influence parasite transmission is a global priority.

Trematode parasites are common disease‐causing agents found in freshwater communities across the globe. Trematodes enter aquatic environments in the feces of definitive hosts, usually a bird or mammal, and hatch into free‐swimming miracidia that infect aquatic mollusks, where they undergo asexual reproduction to produce free‐swimming cercariae (Poulin & Cribb, 2002). Cercariae use chemical and physical cues in the environment to locate their second intermediate host, which include fish, amphibians, or other mollusks (Haas et al., 1994). For these parasites to complete their life cycle, the cercariae form cysts, referred to as metacercariae, in the second intermediate host, which must be consumed by a definitive host (Poulin & Cribb, 2002). Trematode infections may be sensitive to changes in community complexity because trematodes utilize multiple hosts to complete their life cycle and have free‐swimming life stages susceptible to environmental changes (Pietrock & Marcogliese, 2003).

Predators have profound effects on aquatic communities (Sih et al., 1985; Wellborn et al., 1996) and can shape infection dynamics, including those caused by trematodes, through effects on community composition (i.e., density‐mediated effects that change the identity and/or density of community members). For instance, predators may consume free‐swimming parasites such as trematode miracidia or cercariae (Hopkins et al., 2013; Orlofske et al., 2015) or heavily parasitized prey (Gallagher et al., 2019), thus reducing observed parasite infections in hosts and creating “healthier herds” (Packer et al., 2003). Likewise, because many parasites use multiple host species (Woolhouse et al., 2001), certain predators may reduce parasite abundance in vulnerable hosts by acting as hosts themselves (Hatcher et al., 2006). However, predators may also reduce the density of hosts (focal and/or alternative hosts) such that surviving focal hosts experience increased infection loads (Johnson et al., 2009; Rohr et al., 2015; Searle et al., 2011).

Furthermore, predators can mediate parasite transmission by altering host traits (i.e., trait‐mediated effects; Bertram et al., 2013). In response to predators, prey often reduce activity and, consequently, foraging (Kats et al., 1988), which may delay development (Koprivnikar & Penalva, 2015) and increase susceptibility to parasites (Buss & Hua, 2018; Orlofske et al., 2014). Conversely, reductions in host density (and intraspecific competition) mediated by predator consumption may increase developmental rates and reduce parasite infections (Raffel et al., 2010). Like predators, parasites can also have density and trait‐mediated effect on their hosts leading to changes in community‐level processes and the subsequent effects of predators (Buck & Ripple, 2017). For instance, parasites may make their hosts more vulnerable to predation through their effects on host traits (DeBlieux & Hoverman, 2019). The trematode, *Riberoiria ondatrae*, for instance, can cause limb deformities that increase vulnerability to predators (Johnson et al., 2004). Because predator and parasite addition to communities can have substantial and often opposing effects on hosts, it can be challenging to predict how altering community composition influences infection risk.

Predator–prey and host–parasite interactions can result in mortality of the prey or host, but the sublethal effects of these interactions may influence success of surviving hosts later in life. Stressful early life events can impact later fitness in many taxa (Harrison et al., 2011), including amphibians (Chelgren et al., 2006; Van Allen et al., 2010). Trematodes, like those in the genus *Echinostoma*, infect larval amphibians and can cause pathogenic effects depending on infection intensity (Fried et al., 1997; Holland et al., 2007; Schotthoefer et al., 2003), but infections often have little to no effects on their hosts in the absence of other factors (Koprivnikar et al., 2008; Orlofske et al., 2009). However, the effects of trematodes on hosts after metamorphosis are largely unknown. Terrestrial life stages have a disproportionately large impact on amphibian population dynamics (Vonesh & De la Cruz, 2002); therefore, it is critical to investigate the potential sublethal effects of early life conditions on success later in life.

**FIGURE 1:**
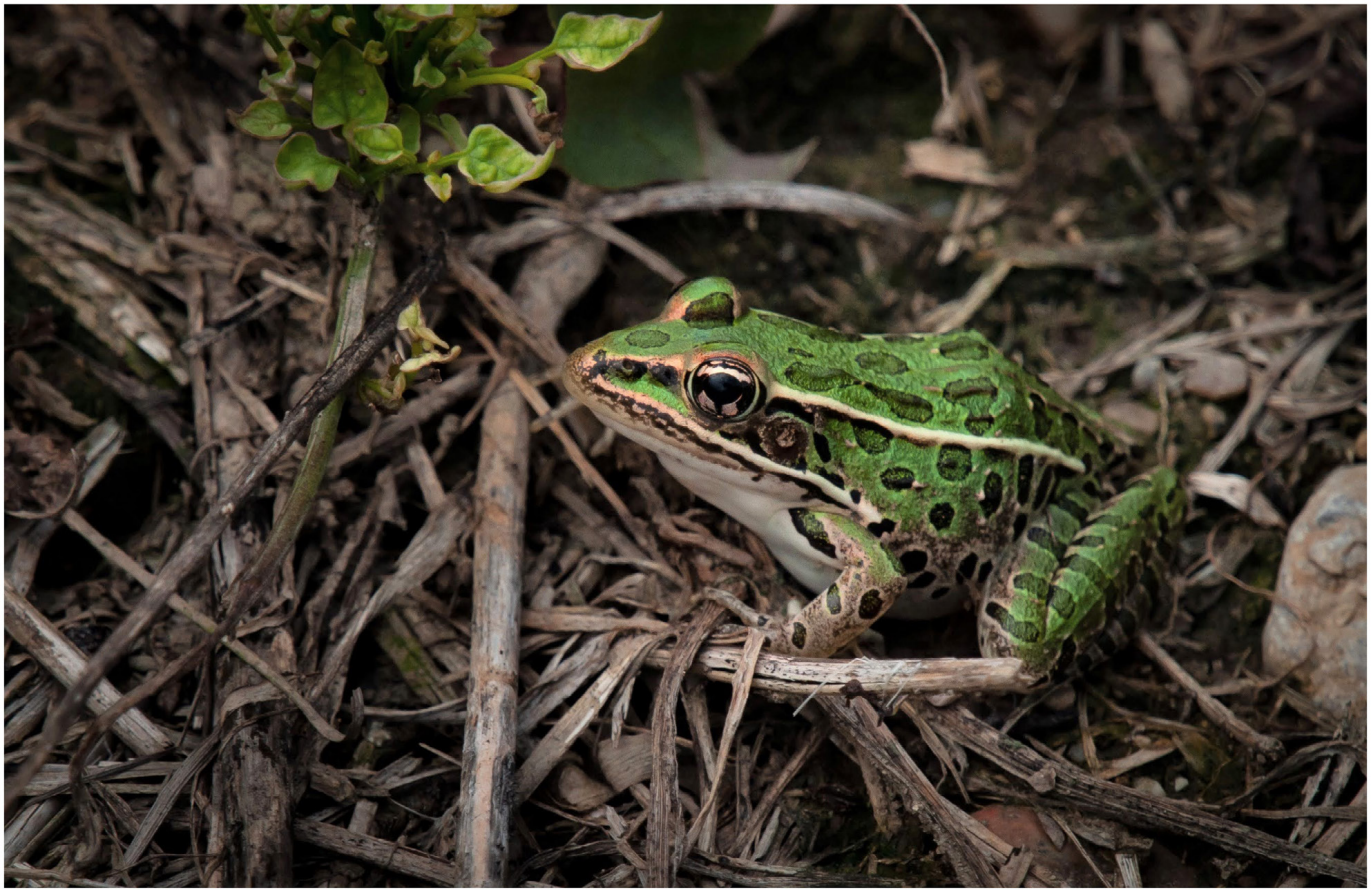
Adult northern leopard frog (*Rana pipiens*). Photo by Mike Wilhelm.

The main objectives of this study were: (1) to evaluate how exposure to potential predators (bluegill [*Lepomis macrochirus*], rusty crayfish [*Orconectes rusticus*], and western mosquitofish [*Gambusia affinis*]) alters the effects of parasites in an amphibian‐trematode model with northern leopard frog (*Rana pipiens*; Figure 1) hosts and *Echinostoma* spp. trematode parasites; (2) to investigate the carryover effects of early life exposure to predators and parasites on juvenile performance in the terrestrial environment; and (3) to model the effects of predators and parasites on population growth (i.e., lambda) of leopard frogs. Because trematode‐induced pathology depends on the intensity of infection (Fried et al., 1997), amphibian‐trematode systems serve as a useful model for understanding how changes in community composition and trophic interactions influence disease (Koprivnikar et al., 2012). Likewise, because amphibians have distinct aquatic and terrestrial life stages, they are excellent models for understanding how early life conditions carryover to later life stages (Boone, 2005; Chelgren et al., 2006; Distel & Boone, 2010; Rumschlag & Boone, 2018; Van Allen et al., 2010).

We hypothesized that the presence of predators would alter the effects of trematode parasites on larval amphibians through direct and indirect pathways and that the effects of the larval environment would carryover into terrestrial life stages, impacting population growth. We predicted that the relative effect of each predator would depend on the simultaneous roles each predator plays in the system. Specifically, we predicted that bluegill, which consume both tadpole (Ade et al., 2010) and snail hosts (Taguchi et al., 2014), and which also serve as alternative hosts for *Echinostoma* spp. (Keeler & Huffman, 2009), would have the strongest negative effects on this system (see information on study system below).

## 2 MATERIALS AND METHODS

### 2.1 Study system

We selected predators for this study that differed in the roles they play within the community. Bluegill can consume small to large tadpoles (Ade et al., 2010), and mosquitofish are more likely to consume small tadpoles (Preston et al., 2012) or cause sublethal injuries (Shulse & Semlitsch, 2014). Though crayfish can reduce survival in amphibians (Ade et al., 2010), tadpoles may be able to escape predation from benthic predators by swimming to the water's surface (Davis et al., 2012). Crayfish and bluegill may also reduce the abundance of snails that serve as parasite hosts (Olsen et al., 1991; Taguchi et al., 2014).

Mosquitofish and bluegill, but not crayfish, can serve as alternative hosts for echinostomes (Keeler & Huffman, 2009). However, this is rarely the case for *Echinostoma* spp. (Orlofske et al., 2015), which primarily encyst in tadpoles or mollusks (Keeler & Huffman, 2009); nonetheless, fish may act as a decoy host in this system. Mosquitofish are efficient predators of cercariae (Orlofske et al., 2015), and bluegill are voracious zooplankton predators (Nowlin & Drenner, 2000; Werner & Hall, 1974), so it is probable that both fish consume cercariae in the water column to reduce parasite abundance. However, because bluegill are selective of prey size and rarely consume small prey items when larger prey items are available (Werner & Hall, 1974), they may preferentially consume large zooplankton over small cercariae. Additionally, large zooplankton that are more common in fishless ponds can also consume cercaria, thereby reducing their density in aquatic systems (Mironova et al., 2019; Schultz & Koprivnikar, 2019).

### 2.2 Animal collection and care

We conducted this experiment in 64 polyethylene mesocosms at Miami University's Ecology Research Center (ERC; Oxford, OH). We filled mesocosms (1.85 m in diameter, 1480 L volume) located at the ERC with 1000 L of city water, 1 kg of mixed leaf litter, and zooplankton/algae inoculates beginning ~5 weeks before predator addition. To inoculate each mesocosm with algae and zooplankton, on four separate occasions over an 8‐day period, we collected water and zooplankton from a local pond using repeated dip‐net sampling in a single bucket and placed 100 ml samples of the pond water mixture into each of the 64 mesocosms. The last inoculation occurred 1 month prior to the addition of any consumers to allow for stabilization of the plankton community. We covered mesocosms with a mesh lid to prevent the colonization of unwanted species. We collected bluegill from a pond at the ERC on 25 March 2017 and rusty crayfish from a stream near Oxford, OH on April 1. Lab‐raised mosquitofish were purchased from Carolina Biological Supply Company (Burlington, NC, USA) on March 31. We added predators to mesocosms on the day of collection.

We collected nine partial northern leopard frog egg masses from a wetland located at Talawanda High School (Oxford, OH) on March 21–22. We held clutches in the laboratory at 22°C until larvae were free‐swimming (Gosner stage 25; Gosner, 1960). In the laboratory, we changed water and fed tadpoles daily. On April 1 (experimental day 0), we mixed tadpoles to homogenize genetic diversity and added 30 tadpoles to each mesocosm.

We collected ramshorn snails (*Planorbella [Helisoma] trivolvis*) from local wetlands between April 11 and 15. We collected snails infected with *Echinostoma* spp. from a wetland at the ERC and uninfected snails from Bachelor Pond in Miami's Natural Areas (Oxford, OH). We held snails individually in aged tap water at 22°C with a 14:10 h light–dark cycle and monitored them daily for cercarial shedding. Snails from Bachelor Pond have never been observed shedding *Echinostoma* spp. cercariae (Miranda Strasburg, personal observation), but we monitored them as a precaution. *Echinostoma* spp. cercariae were identified based on the presence of collar spines (Fried et al., 1997). Morphological similarities prevent species‐level identification of trematodes in the family Echinostomatidae (Johnson & McKenzie, 2009), so we used *Echinostoma* spp., due to widespread distribution of the genus, to refer to all echinostomes (i.e., *Echinostoma* and *Echinoparyhium*) that encysts in kidneys of anurans (Schotthoefer et al., 2003). Snails collected from Bachelor Pond were held for ≥72 h prior to addition to mesocosms to ensure they were not shedding cercariae. Snails received daily water changes and were fed ground algae wafers (Hikari USA Inc.) ad libitum. We added two and three snails to all mesocosms to attempt to control for competition of algal food resources between snails and tadpoles on April 14 and 15 (experimental days 13 and 14), respectively.

### 2.3 Experimental design

We randomly assigned and manipulated predator exposure (two crayfish, bluegill, or mosquitofish [~0.8 predators/m^2^], or no predators) and parasite exposure to *Echinostoma* spp. (via five infected or uninfected snails [~2 snails/m^2^]) with eight replicates per treatment (4 predator treatments × 2 parasite treatments × 8 replicates = 64 mesocosms). We randomly selected half the mesocosms (32 mesocosm) for use in metamorph behaviors trials, while metamorphs from other half were used for in the overwintering portion of the study (see details below). We chose these predator densities as they are within the range of densities in natural communities (Stevenson et al., 1969: approximate bluegill density 0.67–2.02 m^−2^; Smith et al., 2013: mosquito fish 1–32 m^−2^; Lamontagne & Rasmussen, 1993: approximate crayfish density 1–3 m^−2^). We used snail density that are below the densities found in wetlands in the Midwest that serve as common anuran breeding habitats (mean density in wetlands >250 planorbid snails per m^2^; Hentges & Stewart, 2010) due to availability and challenge of screening snails for infection status. At metamorphosis, individuals were randomly assigned to behavioral assays or terrestrial rearing based on mesocosm (four replicates for behavioral assays and four for terrestrial rearing).

To manipulate parasite exposure, we added five infected or uninfected snails (individual diameter: 14.9 mm ± 0.12 [mean ± SE]) to each mesocosm (described above) for the duration of the experiment. To manipulate predator exposure, we added two predators to mesocosms based on treatment assignment (bluegill total length: 88.3 mm ± 0.03, mosquitofish total length: 33.0 mm ± 0.57, crayfish carapace length: 52.3 mm ± 1.61 [mean ± SE]). We matched predators for size prior to their addition to the mesocosms.

### 2.4 Response variables

#### 2.4.1 Snail abundance

To examine how predators and parasites influenced snail density in mesocosms, we counted the number of original snails visible in each mesocosms weekly starting on experimental day 17 and continuing for 7 weeks. Although some snail reproduction did occur in mesocosms, it was not substantial. Juvenile snails were not visible in mesocosms until late in the experiment (>8 weeks); in previous and subsequent experiments conducted by our group, juvenile snails were visible within 2 weeks (Miranda Strasburg, personal observation). The observer was able to differentiate between juvenile snails and the original snails added based on size. We were interested in determining the presence of original snails because those snails would be the only snails actively shedding cercariae within infected ponds, and changes in their abundance could alter trematode effects. The same observer counted the snails each week allowing for relative comparisons of original snails between treatments.

#### 2.4.2 Tadpole behavior

To examine the effects of predators and parasites on tadpole behavior, we monitored tadpole activity weekly in mesocosms starting on experimental day 17. During each observation period, we removed mesocosm lids and allowed tadpoles to acclimate for 30 min before assays were conducted. Twice during each observation period, the same observer counted the number of visible tadpoles and active tadpoles, defined as tadpoles displaying tail movement, throughout the mesocosm; while treatments were unmarked in the field, it was impossible for the observer to be completely blind to treatment because predators were visible in the mesocosms. The entire mesocosms were in view of the observer, which ensured that the tadpoles were not counted twice. Each observation period lasted ~2 min. We averaged counts over the two observation periods to determine the proportion of active tadpoles out of all visible tadpoles.

#### 2.4.3 Metamorph responses

We checked mesocosms daily, removed any metamorph with at least one front limb (Gosner stage 42; Gosner, 1960), and recorded dead metamorphs found within mesocosms, which were included in the calculation of survival to metamorphosis. We weighed surviving metamorphs and determined time to metamorphosis based on tail resorption (Gosner stage 46; Gosner, 1960). We used metamorphs in terrestrial behavioral assays or transferred them to outdoor terrestrial enclosures. Metamorphs not used in the terrestrial portion of this experiment were euthanized using a 1% solution of buffered MS‐222 (tricaine methanesulfonate) and double‐pithed, preserved in 10% neutral buffered formalin for 24 h, and then placed in 75% ethanol until dissection (Miami University IACUC protocol #827). On experimental days 114 and 115, we drained mesocosms and searched through leaf litter for remaining tadpoles (≤2 tadpoles remaining in each mesocosm).

From each of the 32 mesocosms selected for behavior trials, we used the first 10 metamorphs within 48 hr of reabsorbing their tails (Gosner stage 46; Gosner, 1960) and evaluated jumping distance, foraging efficiency, and swimming speed for each individual in behavioral trials. If survival in the mesocosm was <10, we conducted trials with all individuals that reached metamorphosis. First, we placed each metamorph at the end of a plastic tarp and prodded metamorphs to jump eight times. We marked the frog's location at the start of each jump with a dot prior to prodding frog and measured the distance between consecutive marks. If jumping ceased after three consecutive prods, the trial was ended, and those individuals were excluded from behavioral analyses. We determined the average jumping and maximum jumping distance. Next, we placed each metamorph in a plastic container (29.5 cm × 20 cm × 26 cm) lined with a moist paper towel with 20‐3 mm crickets for 15 h; we counted the number of crickets remaining at the end of the trial. Finally, metamorphs were placed in a 1.5 m PVC raceway and encouraged to swim down the raceway. If swimming ceased, the metamorph was gently prodded until swimming resumed; if an individual failed to resume swimming after three successive prods, the trial was ended and excluded from analysis. Of the 294 metamorphs used in behavioral assays, 238 completed all assays; ~6% and ~13% failed to complete jumping and swimming trials, respectively, and two metamorphs escaped during feeding trials. Whether a metamorph completed all trials did not differ between larval treatments (χ^2^ ≤ 4.94, *df* ≤ 3, *p* ≥ .176). After the conclusion of behavioral assays, individuals were euthanized and preserved (described above).

We quantified *Echinostoma* spp. infection load within the kidneys of five randomly selected metamorphs from each mesocosm used in the terrestrial behavioral assays, as is common in mesocosm experiments (Orlofske et al., 2014; Raffel et al., 2010; Rumschlag et al., 2019). We removed each metamorph's kidneys and divided them into two segments. We placed each segment between microscope slides, applied pressure to the slides to reveal cysts, which were then manually quantified. There was evidence of infection with *Ribeiroia ondatrae* in one [parasite‐present, mosquitofish] mesocosm; over 35% of metamorphosing individuals had rear limb deformities, so that mesocosm was removed from all analyses. Although fish can serve has hosts for *Echinostoma* (Keeler & Huffman, 2009), we did not dissect either fish species used to quantify infection load because the bluegill used in this experiment were wild‐caught and may have had prior trematode infections.

#### 2.4.4 Overwintering responses

We reared a subset of metamorphs from the remaining 32 mesocosms in outdoor terrestrial enclosures (2 m × 2 m) at the ERC that contained natural vegetation and arthropods. At the center of each enclosure, we filled a hole (~50 cm deep by ~50 cm diameter) with leaves from a mixed deciduous forest for overwintering and covered it with a wooden board. On the day of tail resorption, we gave each metamorph a toe clip identifier (Phillott et al., 2007). We assigned each metamorph to an enclosure based on larval treatment (2 parasite treatments × 3 predator treatments × 8 replicates = 48 terrestrial enclosures) until each enclosure had five metamorphs (1.25 frogs per m^2^) similar to 2.5 frogs per m^2^ in Distel and Boone (2010); 4 per m^2^ in Boone (2005).

We searched each enclosure and collected all encountered individuals the following spring beginning on May 23, 2018 and continuing every 2–3 days until July 16 when we no longer captured frogs. We weighed and euthanized all frogs as described above (Miami University IACUC protocol #827). To conserve resources, we counted metacercarial cysts in the right kidney only for individuals surviving overwintering because we did not detect any differences in infection load between the right and the left kidneys in metamorphs (*t*‐test between infection load of right and left kidney in metamorphs: *p* = .969, *n* = 75). Additionally, although a right kidney bias has been observed in this system (Johnson et al., 2014), it was not observed in the present study, and we consistently used the right kidney to make relative comparisons of infection load between predator treatments.

### 2.5 Statistical analysis

Prior to statistical testing, we confirmed that all variables meet the assumptions of analysis of variance (ANOVA) using graphical (i.e., histograms and qq‐plots) and statistical methods (i.e., the Shapiro–Wilk test for normality and the Levene's test for homogeneity of variance). Any variables whose distributions deviated considerably from these assumptions were transformed (i.e., mass at metamorphosis, snail abundance, proportion of tadpoles active) or were assessed using non‐parametric models. All analyses were performed in R version 3.6.1.

#### 2.5.1 Snail responses

We used a repeated‐measures analysis of variance (ANOVA) to examine how predators and parasites influenced snail abundance in mesocosms. Snail counts were transformed using a square root transformation.

#### 2.5.2 Amphibian responses

We used a repeated‐measures ANOVA to examine the effects of parasites, predators, and their interactions on tadpole behavior using the average proportion of tadpoles active (arc sin square root transformation) each week as a repeated measure. We analyzed the effect of treatment on the proportion of visible tadpoles that were active to control for treatment effects on survival that would influence the number of tadpoles visible. We used a generalized linear model (GLM) with a binomial distribution to test the effects of parasites, predators, and their interaction on survival to metamorphosis and the number of animals that reached metamorphosis but were found dead in the mesocosm with the number measured for each mesocosm. The effects of parasites, predator exposure, and their interaction on time to metamorphosis and mass at metamorphosis were analyzed using ANOVAs with mesocosm as the experimental unit. Mass was log‐transformed to meet the assumption of normality. We used ANOVAs to determine if the sublethal effects of early life conditions influenced terrestrial behavior (maximum jumping distance, average jumping distance, and swimming speed, number of crickets consumed). Likewise, because the body condition of metamorphs may influence their behavior in the terrestrial environment, we examined if mass at metamorphosis (log transformed) was correlated with behavioral responses using a Pearson correlation test.

To determine the influence of predators on parasite infection load in metamorphs, we used a generalized linear mixed‐effects model (GLMM) with a Poisson distribution. To prevent pseudoreplication, mesocosm was used as a random effect to account for individuals that were reared together. We tested for overdispersion using “dispersion_glmer” function in “blmeco” R package, and the resulting value (1.1) did not exceed the threshold value (1.4) necessary to suggest overdispersion (Korner‐Nievergelt et al., 2015). Because cysts were absent in the animals from the no‐parasite treatment, those mesocosms were not included in these analyses. We repeated this analysis when we analyzed the parasite infection load (in the right kidney only) of the individuals that survived through overwintering but used terrestrial enclosure as a random effect. There was no evidence of overdispersion (1.00 < 1.4). We determined if parasite infection load was correlated with the average number of original snails visible in each mesocosm throughout the experiment using a GLMM with a Poisson distribution. For this analysis we used infection load in the right kidney only and included mesocosm and infection sampling time (i.e., at metamorphosis or after overwintering) as random effects.

#### 2.5.3 Overwintering responses

Two enclosures (both from the no predator treatment but differing in their parasite treatment) had no animals survive due apparently to shallow refugia (<30 cm) and were, therefore, excluded from analysis. We used a GLM with a binomial distribution to test the effects of parasites, predators, and their interaction on survival post‐overwintering with the number that survived measured for each terrestrial enclosure. We tested for the effects of larval parasites, predators, and their interaction on change in mass from metamorphosis until after overwintering using ANOVA with terrestrial enclosure as the experimental unit. We also used a GLMM with a binomial distribution to determine the effects of mass at metamorphosis on terrestrial survival and a linear mixed‐effects model (LMM) to determine if mass at metamorphosis influenced final mass with enclosures included as a random effect to control for pseudoreplication. We used linear regression to determine if total parasite load was correlated with change in mass from metamorphosis through overwintering.

### 2.6 Population growth model

To determine if the effects of trematodes and bluegill on tadpole survival could influence population growth in northern leopard frogs, we developed a female‐based Lefkovitch annual projection matrix using three life‐history stages (Caswell, 2000): pre‐juvenile (embryo, tadpole, and overwintering metamorphs), juvenile, and reproductive adult (as in Biek et al., 2002). This model simulated expected survival at a yearly timestep. We used the following projection matrix to model northern leopard frog populations under control conditions:

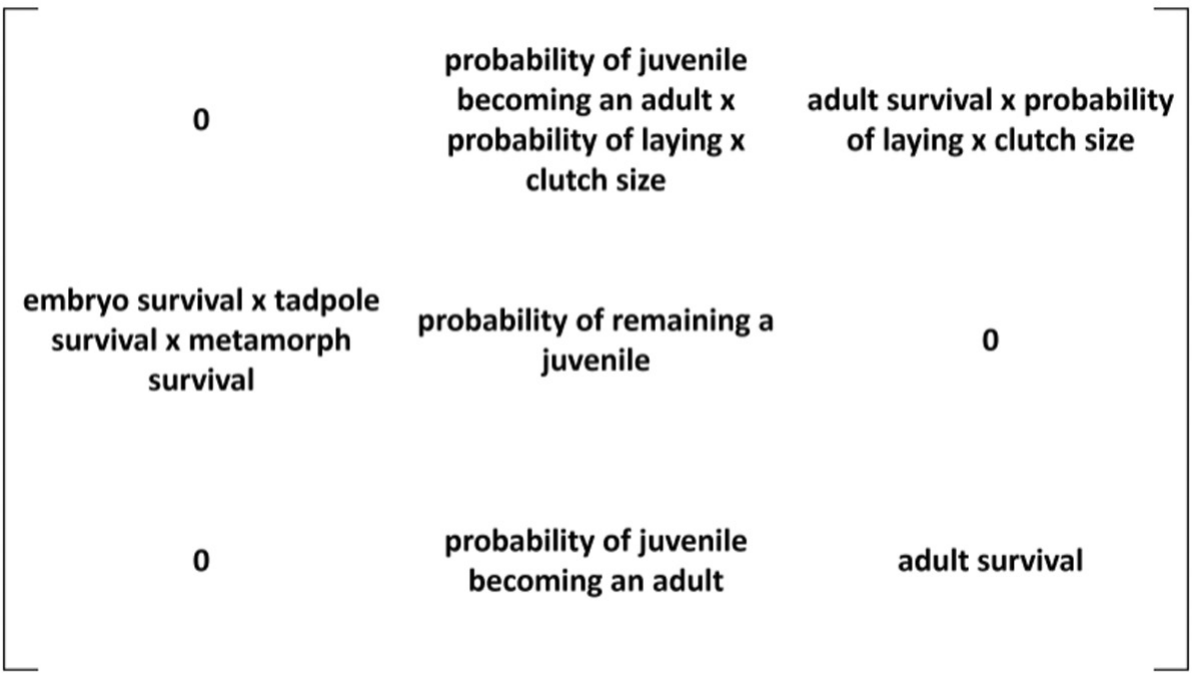

To understand the direct effects of parasites and bluegill on population growth, we modeled dynamics under three scenarios based on experimental data: no exposure to parasites or bluegill, exposure to parasites, exposure to bluegill, and exposure to both parasites and bluegill. Under the latter three scenarios, we reduced tadpole survival by 23%, 40%, or 30% based on the influence of parasites, bluegill, or their combined effects, respectively, in the present study. Because reductions in tadpole survival mediated by both parasites and bluegill increased mass at metamorphosis in our experiment and larger anurans often reach reproductive maturity earlier (Smith, 1987), we modeled the sublethal effects of bluegill and parasites on leopard frogs by reducing the average time it took for individuals to transition from juveniles to adults, while retaining the same pre‐juvenile survival used in our control model. Northern leopard frogs typically reach sexual maturity in 2–3 years (Force, 1933; Gilbert et al., 1994), but have been observed to breed 1‐year post‐hatching (Ryan, 1953). Indeed, nearly 10% of the animals that survived overwintering in this study had reached a snout vent length (SVL) of ≥60 mm, which is considered sexually mature for female leopard frogs (Gilbert et al., 1994; an additional 14% were ≥55 mm in length), and there were individuals with eggs and convoluted oviducts (both signs of maturity in anurans; Harper & Semlitsch, 2007). In our model estimating the indirect, sublethal effects of bluegill and parasites on population growth, we reduced the minimum age at which northern leopard frogs reach sexual maturity from 2 years to 1 year, thus altering the average time to maturity from 2–3 years (as in our direct effects models) to 1–3 years. This led to an increase in the average probability that juveniles transitioned to adults from 0.062 to 0.192 and reduced the probability that they remained juveniles from 0.293 to 0.162 (Table 1). We determined vital rates to use in our models from the literature (Table 1; as in Biek et al., 2002; Rumschlag & Boone, 2018). We calculated the mean finite rate of increase (λ) for northern leopard frog populations for each scenario after 2000 iterations where vital rates were randomly selected from a distribution. We used a log‐normal distribution for clutch size and β‐distributions for all other vital rates using means and standard deviations based on Biek et al. (2002). To better understand how each life stage contributes to λ, we followed our calculation of λ with elasticity analyses for the annual project matrix representing northern leopard frog population with no exposure to parasites and bluegill and no change in reproductive maturity (i.e., the control scenario) using the mean vital rates (Table 1). Elasticity analysis shows the relative contribution of an individual stage to λ (De Kroon et al., 2000). We completed the population modeling exercises in R version 3.6.1 using code adapted from Stevens (2009) and Biek et al. (2002).

**TABLE 1:** Vital rates and transition probabilities used in the stage‐structure matrices.

Vital rate	Mean (SD)	Species
Embryo survival	0.700 (0.049)^a,b^	*Rana sylvatica*
Larval survival	0.036 (0.012)^a,b^	*R. sylvatica*
Metamorph survival	0.750 (0.165)*	*R. pipiens*
Juvenile survival	0.355 (0.093)^b^	*R. aurora*
Juvenile to juvenile		
Average maturity	0.293 (0.036)^b,c^	*R. aurora*
Early maturity	0.162 (0.036)^b,c^	*R. aurora*
Juvenile to adult		
Average maturity	0.062 (0.056)^b,c^	*R. aurora*
Early maturity	0.192 (0.056)^b,c^	*R. aurora*
Adult survival	0.686 (0.133)^b^	*R. aurora*
Probability of laying	1^b^	
Clutch size	2659 (480)^d^	*R. pipiens*
Age at sexual maturity (yr)	1 to 3^d,e,f^	*R. pipiens*

*Note*: ^a^Berven, 1990, ^b^Biek et al., 2002, ^c^Crouse et al., 1987, ^d^Gilbert et al., 1994, ^e^Force, 1933, ^f^Ryan, 1953, *This experiment.

## 3 RESULTS

### 3.1 How do predators impact host–parasite interactions in aquatic environments?

**FIGURE 2:**
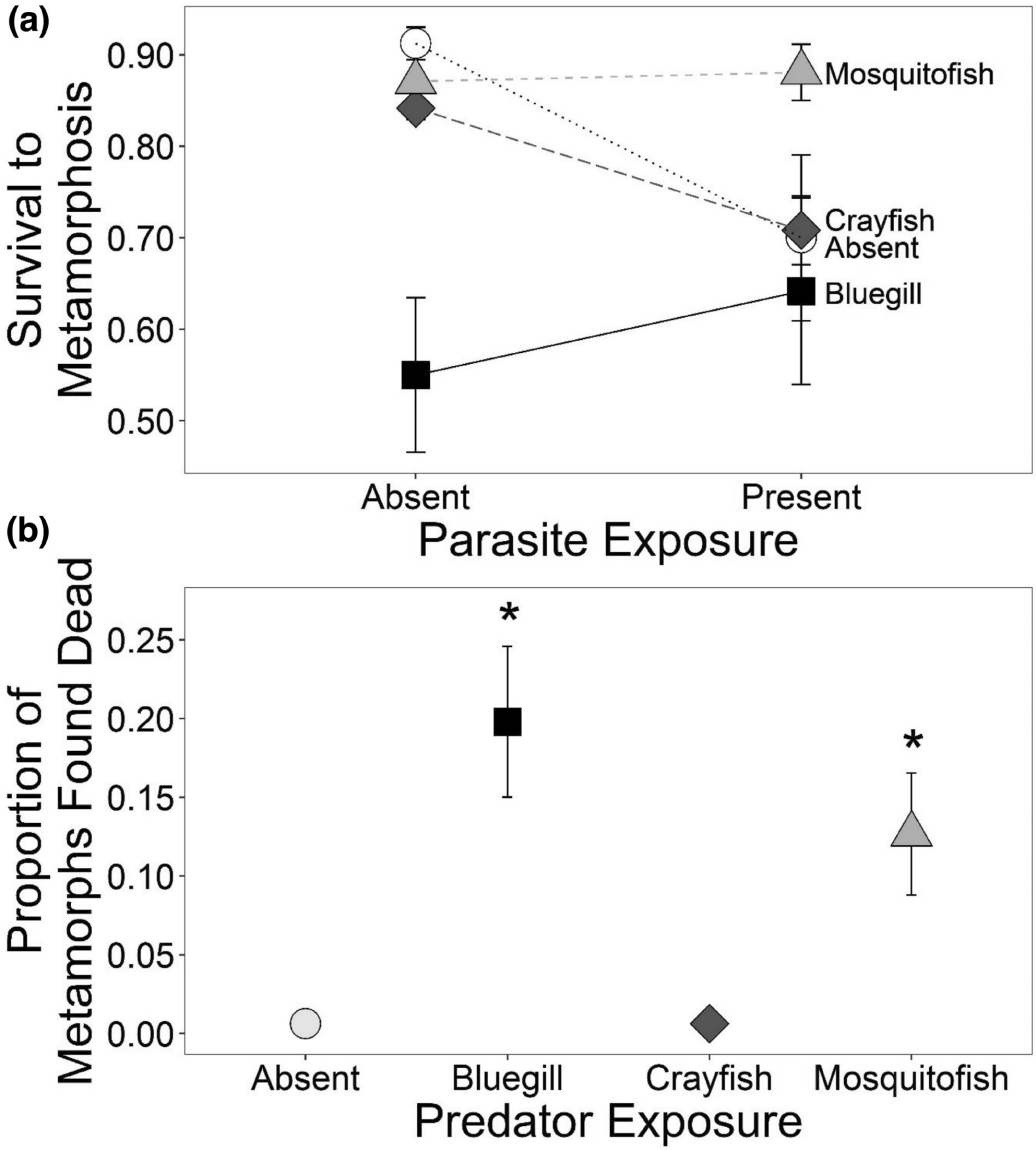
Influence of treatment on survival in the aquatic environment. (a) Proportion of leopard frog tadpoles exposed to predator treatments (none, bluegill, crayfish, or mosquitofish) and parasite treatments (absent, present) that survived to metamorphosis. (b) Proportion of individuals exposed to predator treatments that died in mesocosms after reaching metamorphosis. Plotted values are means ±1 SE. Asterisks indicate differences from the no predator treatment based on Dunnett's pairwise comparisons.

The influence of predators on host–parasite dynamics in this system depended greatly on predator identity; generally, bluegill had strong effects on multiple responses, whereas the effects of mosquitofish and crayfish were negligible or absent. For survival to metamorphosis, the effect of predator varied with parasite exposure, such that bluegill and mosquitofish moderated the effects of parasites (Table 2). When crayfish were present or predators were absent, parasites reduced survival to metamorphosis by 23% and 15%, respectively, yet parasites did not reduce survival when either fish was present (Figure 2a). Survival to metamorphosis was high in the presence of mosquitofish regardless of parasite presence and was lowest in the presence of bluegill regardless of parasite presence (Figure 2a). Further, fish presence significantly increased metamorph mortality within mesocosms (Table 2). Bluegill and mosquitofish resulted in mortality of 20% ± 5 and 12% ± 3 (mean ± SE) of northern leopard frogs that reached metamorphosis (e.g., Gosner 42), respectively, while <1% of metamorphs (three total in each treatment) died in mesocosms with crayfish or no predators (Figure 2b).

**TABLE 2:** Summary of generalized linear model (GLM) with binomial distribution for survival to metamorphosis, proportion of metamorphs found dead in mesocosm (tadpoles that reached Gosner stage 42 but died before removal from mesocosm), and survival through overwintering.

Response variable	Source of variation	*df*	Wald χ^2^	*p*
Survival to metamorphosis	Parasite	1	31.12	**<.001**
	Predator	3	104.98	**<.001**
	Parasite by predator	3	39.58	**<.001**
Metamorphs dead in mesocosm	Parasite	1	0.12	.726
	Predator	3	63.98	**<.001**
	Parasite by predator	3	6.86	.077
Survival through overwintering	Parasite	1	1.32	.248
	Predator	3	4.80	.187
	Parasite by Predator	3	1.25	.740

*Note*: Significant effects (α ≤ 0.05) are in bold text.

**FIGURE 3:**
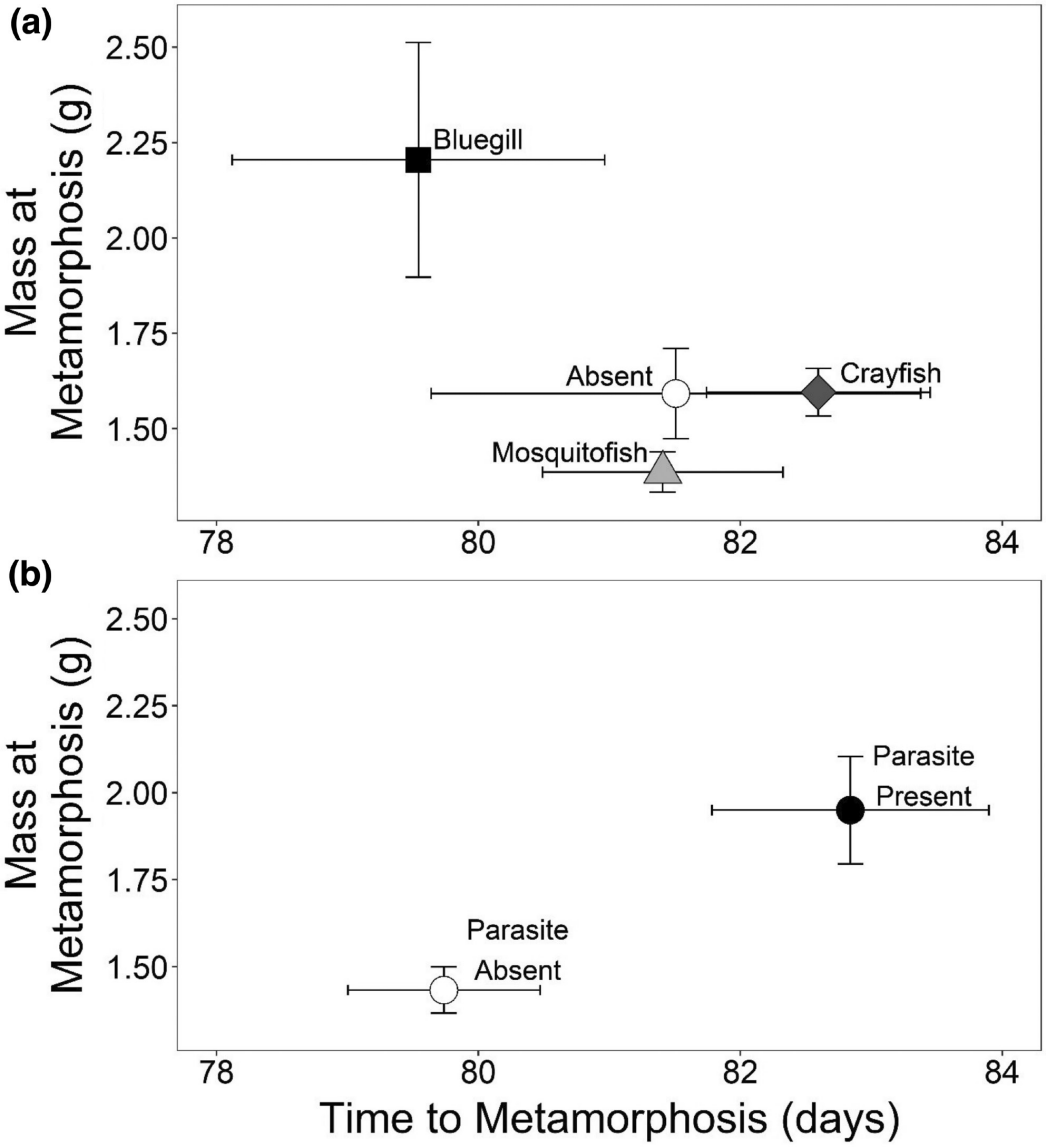
Mass at metamorphosis and larval period for leopard frogs exposed to (a) different predator treatments (none, bluegill, crayfish, or mosquitofish) and to (b) different parasite treatments (absent, present). Plotted values are means ±1 SE.

**FIGURE 4:**
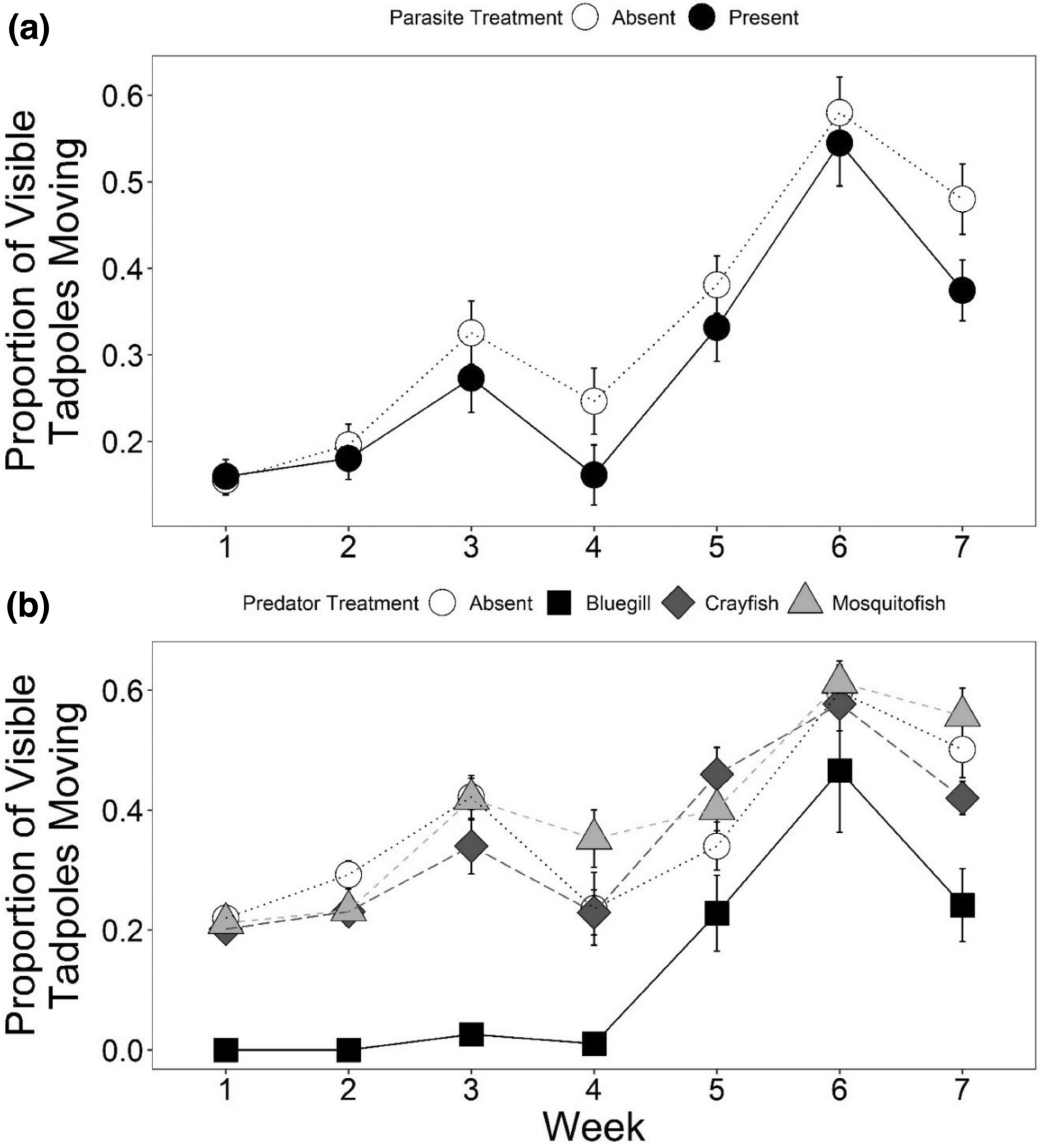
The average proportion of leopard frog tadpoles moving in each mesocosms exposed to (a) different parasite treatments (absent, present); and to (b) different predator treatments (none, bluegill, crayfish, or mosquitofish) for 7 weeks. Plotted values are means ±1 SE.

Predator treatment, specifically the presence of bluegill, also had significant sublethal effects on tadpoles (Table 3). Bluegill presence led to 35% larger mass at metamorphosis relative to predator controls (Dunnett's test *p* = .005), without significant impacts on time to metamorphosis (Figure 3a). Conversely, parasites significantly increased time to metamorphosis by ~2 days, which was associated with a 33% increase in mass at metamorphosis relative to mesocosms without parasites (Table 3; Figure 3b). Bluegill and parasites, though less strongly than bluegill, each significantly reduced tadpole activity relative to the control (Table 4; Figure 4). After week 4, there was an increase in tadpole activity across treatments (Figure 4).

**TABLE 3:** Summary of ANOVAs for mass at metamorphosis, time to metamorphosis, metamorph behaviors, and change in terrestrial mass.

Response variable	Source of variation	*df*	*F* value	*p*
Mass at metamorphosis	Parasite	1, 54	16.12	**<.001**
	Predator	3, 54	4.69	**.006**
	Parasite by predator	3, 54	1.50	.225
Time to metamorphosis	Parasite	1, 54	5.81	**.019**
	Predator	3, 54	0.52	.670
	Parasite by predator	3, 54	0.70	.555
Swimming speed	Parasite	1, 230	0.02	.901
	Predator	3, 230	2.99	**.032**
	Parasite by predator	3, 230	0.19	.901
Maximum jumping distance	Parasite	1, 230	31.71	**<.001**
	Predator	3, 230	12.91	**<.001**
	Parasite by predator	3, 230	9.93	**<.001**
Average jumping distance	Parasite	1, 230	27.94	**<.001**
	Predator	3, 230	13.15	**<.001**
	Parasite by predator	3, 230	9.36	**<.001**
Number of crickets consumed	Parasite	1, 230	0.01	.922
	Predator	3, 230	3.28	**.022**
	Parasite by predator	3, 230	3.28	**.022**
Change in terrestrial mass	Parasite	1, 38	2.10	.155
	Predator	3, 38	2.83	.051
	Parasite by predator	3, 38	2.19	.104

*Note*: Mesocosm was the experimental unit for mass and time to metamorphosis, and terrestrial enclosure was the experimental unit for change in terrestrial mass. Significant effects (α ≤ 0.05) are in bold text.

**TABLE 4:** Summary of repeated‐measures ANOVA for the number of original snails visible and the average proportion of tadpoles moving within mesocosm during the first 7 weeks of the experiment.

Response variable	Source of variation	*df*	*F* value	*p*
Number of snails	**Between subjects**			
	Parasite	1, 55	1.06	.307
	Predator	3, 55	16.70	**<.001**
	Parasite by predator	3, 55	1.00	.399
	**Within subjects**			
	Time	6, 330	8.62	**<.001**
	Time by parasite	6, 330	1.00	.427
	Time by predator	18, 330	1.84	**.020**
	Time by parasite by predator	18, 330	0.58	.913
Average proportion of tadpoles moving	**Between subjects**			
	Parasite	1, 55	5.12	**.022**
	Predator	3, 55	44.15	**<.001**
	Parasite by predator	3, 55	0.26	.855
	**Within subjects**			
	Time	6, 330	51.01	**<.001**
	Time by parasite	6, 330	0.96	.450
	Time by predator	18, 330	2.23	**.003**
	Time by parasite by predator	18, 330	1.12	.330

*Note*: Significant effects (α ≤ 0.05) are in bold text.

**FIGURE 5:**
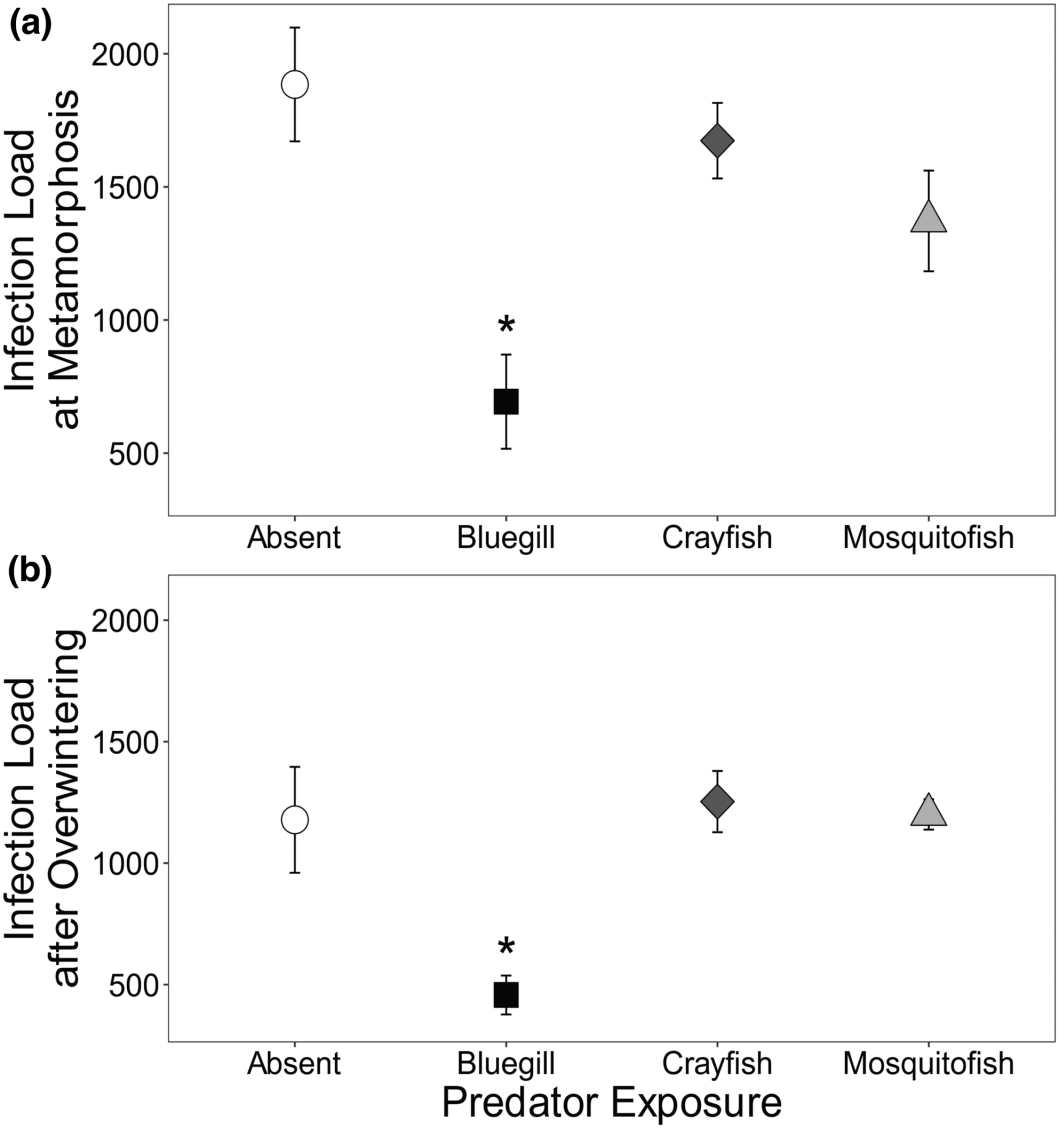
Average parasite load of frogs exposed to predator treatments (none, bluegill, crayfish, or mosquitofish) (a) at metamorphosis or (b) after overwintering. Plotted values are means of right kidneys only ±1 SE. Asterisks indicate differences from the no predator treatment based on Dunnett's pairwise comparisons.

Parasite infection load ranged from 33 to 8589 cysts per metamorph (2822 ± 207.7 [mean ± SE]) and was reduced in predator treatments (χ^2^ = 12.98, *df* = 3, *p* = .005; Figure 5a). Bluegill exposure significantly reduced infection load by 66% on average relative to the predator control (Generalized Linear Mixed Model [GLMM] with Poisson distribution, *Z* = −3.19, *p* = .001). Although exposure to mosquitofish and crayfish reduced infection load on average, these reductions were not statistically significant (GLMM with Poisson distribution, *Z* = −0.65 and −0.18, *p* = .44 and .85, respectively). The average number of original snail visible throughout the experiment also significantly influenced infection load (χ^2^ = 12.32, *df* = 1, *p* < .001). There was a positive relationship between average number of snails visible and total infection load (Generalized Linear Mixed Model [GLMM] with Poisson distribution, *Z* = 3.51, *p* < .001).

**FIGURE 6:**
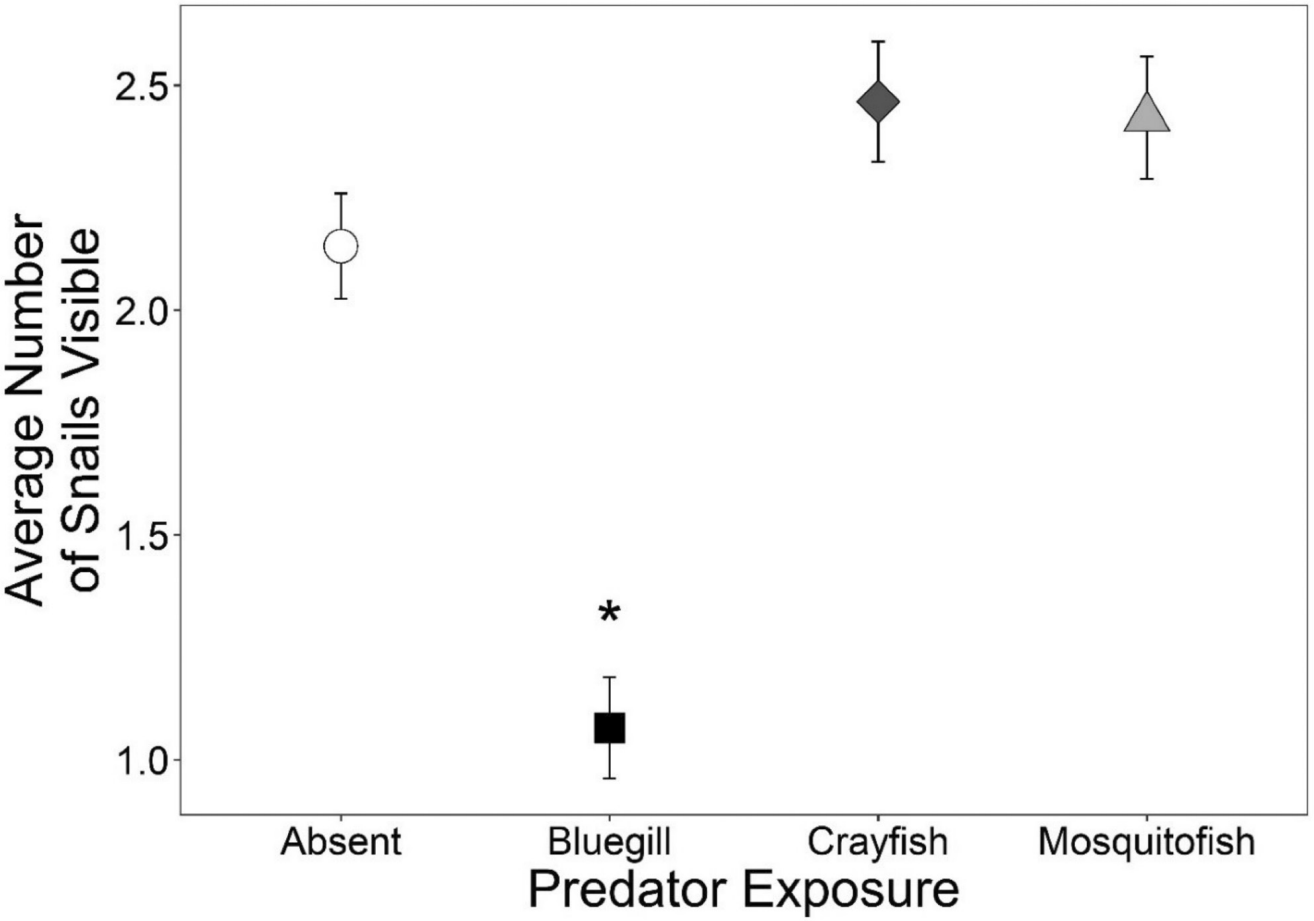
Average number of original snails visible in mesocosms exposed to predator treatments (none, bluegill, crayfish, or mosquitofish). Plotted values are means ±1 SE. Asterisks indicate differences from the no predator treatment based on Dunnett's pairwise comparisons.

Snail abundance (of snails added at the beginning of the study) was significantly influenced by predator but not parasite treatment (Table 4). Bluegill reduced the number of snails visible throughout the experiment relative to the no predator treatment; however, mosquitofish and crayfish did not influence the number of snails visible (Figure 6).

### 3.2 Do the effects of aquatic predators and parasites carryover to the terrestrial life stage?

**FIGURE 7:**
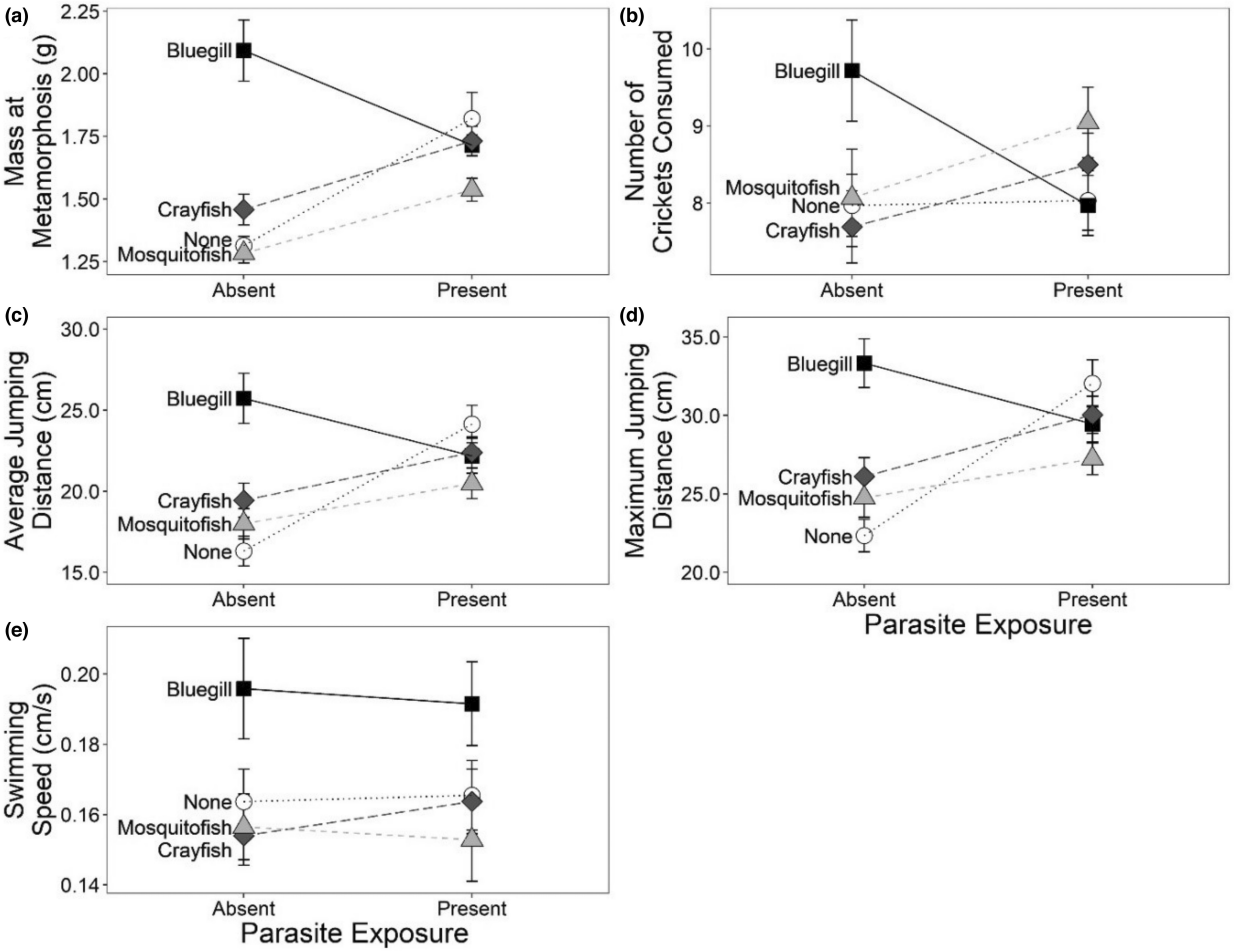
Influence of predator (none, bluegill, crayfish, or mosquitofish) and parasite treatment (absent, present) on (a) mass at metamorphosis, (b) average jumping distance, (c) maximum jumping distance, (d) swimming speed, and (e) number of crickets consumed by leopard frog metamorphs. Figure 7a Only shows the mass at metamorphosis for individuals that were used in the behavioral trials. Plotted values are means ±1 SE.

**FIGURE 8:**
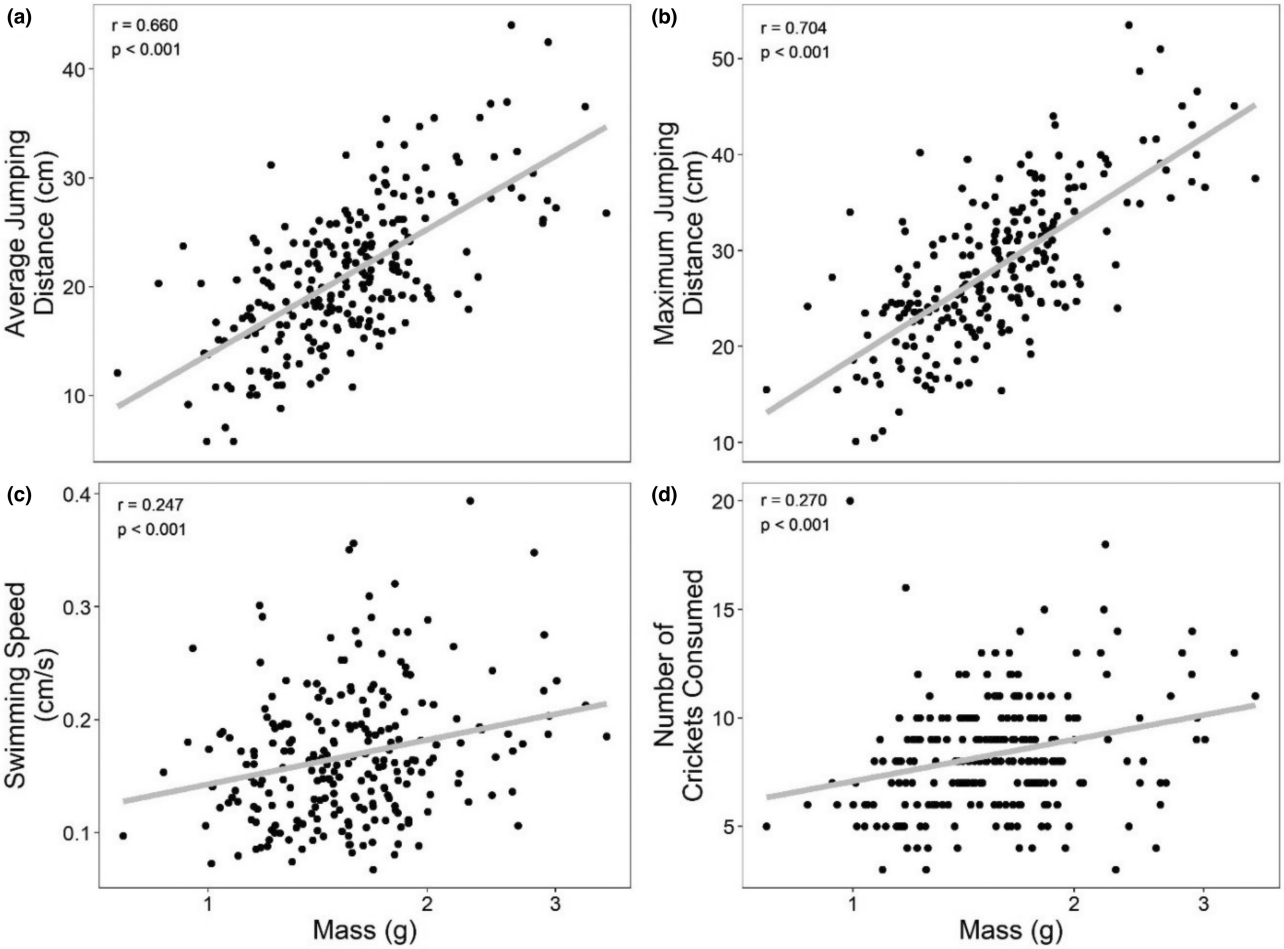
Relationship between mass at metamorphosis and (a) average jumping distance, (b) maximum jumping distance, (c) swimming speed, and (d) number of crickets consumed within 15 h. *X*‐axis is log transformed. Linear regression lines of best fit show the nature of relationship.

The sublethal effects of predators and parasites on larval development influenced metamorph behavior in the terrestrial environment. Parasite and predator exposure in the larval environment interacted to influence maximum and average jumping performance as well as the number of crickets consumed (Table 3). In these cases, the mass at metamorphosis corresponded with the relative response of jumping and feeding behaviors, such that larger frogs jumped farther and ate more crickets (a covariate was not used in analysis because size differences were not distributed across treatment such that size differences were confounded by treatment; Figures 7a–d). Mass at metamorphosis was positively correlated with performance for jumping, swimming speed, and feeding (Pearson correlation test, *p* < .001; Figure 8). Predator treatment alone significantly affected swim speed; metamorphs reared with bluegill swam significantly faster than those from other predator treatments (Figure 7e).

Although parasite and predator exposure influenced mass at metamorphosis and terrestrial behavior, they did not affect overwinter survival (Table 2; Figure S1a), which was high (85 ± 2% [mean ± SE]). Metamorphs gained an average of 10.7 ± 0.4 g (mean ± SE) after overwintering. There was a marginal effect of larval aquatic predators on change in mass (Table 3; Figure S1b), but post hoc tests revealed no significant differences between groups (Dunnett's contrasts *p* > .069). Although mass at metamorphosis was correlated with final mass (LMM, *F*_1,188_ = 50.69, *p* < .001), metamorph mass did not affect survival through overwintering (GLMM with binomial distribution, *Z* = 0.022, *p* = .983).

All metamorphs that survived to overwintering maintained their parasite infections. The metacercarial cyst abundance after overwintering ranged from 14 to 2997 cysts per animal (1003 ± 71.6 [mean ± SE]; Figure 5b). As with infection load at metamorphosis, larval predator exposure significantly reduced parasite infection load post‐overwintering (χ^2^ = 20.56, *df* = 3, *p* < .001) for frogs exposed to bluegill (GLMM with Poisson distribution, *Z* = −2.93, *p* = .003); however, all treatments showed lower parasite infection loads when compared with the infection loads of metamorphs. Additionally, parasite infection load was not correlated with change in terrestrial mass (*R* ^2^ = 0.004, *F*_1,95_ = 0.39, *p* = .534).

### 3.3 Do effects of aquatic predators and parasites influence population growth (λ)?

**FIGURE 9:**
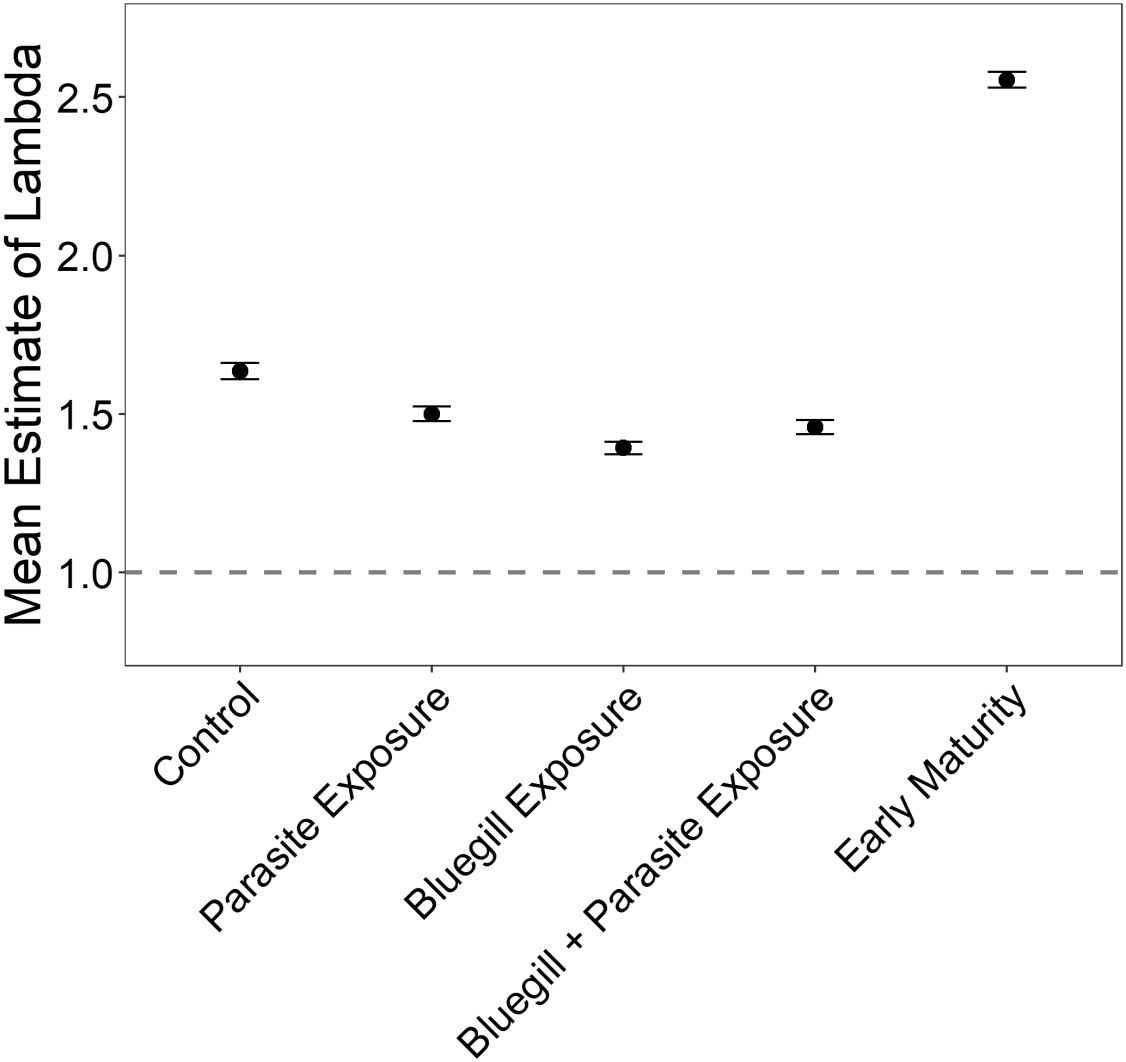
Mean λ (the finite rate of increase of population growth) values with 95% confidence intervals under experimental conditions. Parasite, bluegill, and their combined exposure represent scenarios where population growth is influenced by treatment effects on metamorph survival (i.e., lethal effects). The fast maturity scenario represents an earlier time to first reproduction resulting from the effects of parasites and bluegill on body condition (i.e., sublethal effects).

Parasites and bluegill have the potential to affect the finite rate of increase of population growth, λ, through both lethal and sublethal effects. In the absence of both parasites and bluegill, our model indicated a mean λ of 1.63, indicating a growing population (λ > 1 is indicative of population growth; Figure 9). When survival to metamorphosis is reduced by 23% and 40%, as observed in this study with parasite and bluegill exposure, mean λ decreased by 8% and 15% respectively, with the population still expected to increase (Figure 9). When both parasites and bluegill were present in mesocosms, survival to metamorphosis was only reduced by 30%, as such mean λ increased with both parasite and bluegill exposure compared with bluegill exposure alone (Figure 9). However, reducing time to sexual maturity, which can occur when individuals reach metamorphosis at a large size, had a greater effect on mean λ than the direct effects of bluegill and parasites on survival and led to an increase in mean λ by 59% (Figure 9). The elasticity analysis revealed that changes in pre‐juvenile survival have the greatest impact on λ relative to changes in other matrix elements (Table 5).

**TABLE 5:** Elasticity values for projection matrices representing control northern leopard frog *(Lithobates pipiens)* populations.

Life stage transition	Pre‐juvenile	Juvenile	Adult
Pre‐juvenile	0	0.221	0.134
Juvenile	0.352	0.071	0
Adult	0	0.138	0.084

## 4 DISCUSSION

Ecological communities form a complex network of interacting species, and changes in this network can substantially impact interactions between hosts and parasites. However, not all changes in community composition have equal impacts. Our study shows predators, such as bluegill, that play multiple roles within a community, alter the outcome of parasite infection through both direct effects on hosts and indirect food web effects. Additionally, our results show that sublethal effects of natural enemies on prey/host body condition may have long‐term fitness benefits for surviving individuals and their population.

### 4.1 Bluegill predators had the greatest impact on the community and altered the impact of parasites

The presence of fish, like the presence of many top predators (Ripple et al., 2014), can fundamentally shape the food webs of natural systems (Wellborn et al., 1996). Bluegill, the largest and most efficient predator in our study, had the strongest impact on the community and their effects reduced infection load and increased body condition of frogs that survived to metamorphosis. Only bluegill significantly reduce tadpole density in the absence of parasites, suggesting that tadpoles were able to escape predation by crayfish and mosquitofish. Despite their effect on tadpole density, bluegill exposure did not increase infection load in surviving tadpoles, as observed by others (Rohr et al., 2015). Instead, by reducing the abundance of snail hosts likely through consumption, which led to reduced parasite loads in metamorphs and juveniles that overwintered, bluegill reduced trematode transmission to tadpoles. Although bluegill can serve as hosts for trematodes, this is rarely the case with *Echinostoma* spp. (Orlofske et al., 2015). However, given that trematodes rely on physical and chemical cues to locate their hosts (Combes et al., 1994; Haas, 2003), parasites may have responded to bluegill particularly given that tadpole activity was reduced with bluegill exposure, which may have made tadpoles less detectable. Across all treatments, we observed an increase in tadpole activity after week 4, which is likely attributable to changes in tadpole behavior with development, as larval anurans increase activity as they develop until they near metamorphosis (Cheron et al., 2021).

The presence of bluegill and mosquitofish also negated the impact of parasites on hosts: parasites negatively impacted anuran survival only in the predator control and crayfish treatments. Mosquitofish reduced the impact of parasites on host survival even though infection load was similar to predator controls, possibly through their indirect effects on the surrounding community which may have increased algal food resources. By consuming zooplankton, mosquitofish may have increased the algal resource base available for tadpoles (as in Preston et al., 2012), such that the availability of food resources offset the negative effects of parasites on survival. On average, mosquito fish also reduced parasite load in metamorphs compared with the predator controls, though this effect was not statistically significant. Nonetheless, this small reduction in parasite load, possibly mediated by the consumption of cercariae by mosquito fish (Orlofske et al., 2015), may have contributed to the reduced effects of parasites on survival in the presence of mosquito fish. In addition to their observed effect on tadpole abundance, bluegill also can reduce zooplankton abundance (Nowlin & Drenner, 2000), which can lead to algal blooms, further releasing surviving tadpoles from competition. As with mosquitofish, we did not observe an additive effect of parasites on survival with bluegill though metamorph survival was considerably lower in mesocosms with these efficient predators. Consistent with other host–parasite systems (Ezenwa, 2004; Knutie et al., 2017), we found that potential increases in resource availability, mediated by reduction in competition, may reduce the direct negative effects of parasites on hosts. Additionally, the morphological changes associated with metamorphosis increased vulnerability to fish predators, suggesting that the importance of predators may change depending on prey condition (Murray, 2002; Tucker et al., 2016). Further research is needed to understand how changes in food web structure mediated by predator presence indirectly influence interactions between hosts and parasites, as we did not directly measure algal or zooplankton abundance in the present study.

Parasites reduced survival to metamorphosis only in fishless communities emphasizing the importance of community composition in regulating parasite effects. Fishless, ephemeral wetlands are the preferred breeding habitat for many amphibian species including northern leopard frogs (Kendell, 2002). However, over recent decades, the abundance and quality of ephemeral wetlands across the landscape have declined due to anthropogenic impacts including climate change, draining for agricultural purposes, and introduction of exotic species (Calhoun et al., 2017). These changes may force amphibians to breed in suboptimal habitat, thereby altering their interactions with parasites. Changes in wetland permanence can also alter the presence of freshwater snails (Hoverman et al., 2011; Urban & Roehm, 2018); our results show that bluegill presence influenced snail abundance and consequently impacted the abundance of trematodes in subsequent hosts. Here, we ultimately demonstrated that the influence of parasites on hosts is not consistent across all communities. In the presence of certain predator species (i.e., crayfish), parasites reduced host survival, but this impact was absent in the presence of other predators (i.e., bluegill and mosquito fish). Because predators can have both density‐ and trait‐mediated effects, as observed in our present study, their effects on host–parasite interaction can be highly variable (Lopez & Duffy, 2021), emphasizing the need for experiments that mimic the complexity of natural ecological communities.

### 4.2 The sublethal effects of predators influenced success later in life

Conditions of early development may impact the performance and survival of organisms later in life (Harrison et al., 2011). In the present experiment, conditions in the larval environment resulted in differential size at metamorphosis and parasite load, which set the stage for varying success in the terrestrial environment. Although predators had no effect on time to metamorphosis, tadpoles exposed to bluegill were larger at metamorphosis. The observed and expected thinning of tadpoles and zooplankton populations (Nowlin & Drenner, 2000), respectively, by bluegill predators can create a food‐rich environment (Morin, 1983). Parasites also increased mass at metamorphosis by prolonging the larval period and reducing intraspecific competition. Although reproduction in snails was minimal throughout this experiment (Miranda Strasburg, personal observation), it is also possible that parasites reduced interspecific competition between tadpoles and snails by limiting snail reproduction, as trematodes castrate their snail hosts (Marchand et al., 2020). Further, other studies suggest that increased mass with parasite exposure may be attributable to cannibalism of decomposing conspecifics (Marino, 2016), which likely provide a nutrient‐rich food source for surviving tadpoles. By remaining in the aquatic environment despite the potential direct negative effects of bluegill and parasites on survival, surviving individuals were able to take advantage of high food conditions and attain a larger size at metamorphosis.

Regardless of the mechanisms driving increased mass at metamorphosis, larger individuals outperformed smaller individuals in terrestrial behavior assays. Although the amphibian host must be consumed to transmit the parasite to the definitive host, a large host may benefit the parasite, as individuals with higher body conditions are better suited to endure parasite infections (Beldomenico & Begon, 2010; Vollset, 2019), to survive stressful periods such as overwintering (Rumschlag & Boone, 2018), and to return to natal ponds to breed where they may be most susceptible to definitive hosts. Likewise, smaller tadpoles are more vulnerable to predation by aquatic invertebrates (Brodie & Formanowicz, 1983), so reaching a large size may ensure successful metamorphosis and increase the likelihood of consumption by definitive hosts for the parasites.

Additionally, in the present study, individuals maintained larger body sizes through overwintering, which could benefit populations of anuran hosts as larger individuals reach sexual maturity faster (Smith, 1987) and are generally more fecund (Gilbert et al., 1994). Although the long‐term effects of larval trematodes (i.e., metacercariae) on anurans are largely unknown, this study suggests that tadpole infection by these parasites does not hinder their host's success in the terrestrial environment regardless of infection load because growth was not influenced by infection load and overwintering survival was consistent across all treatments. Given that trematodes are increasing in some areas of the United States (Johnson & McKenzie, 2009), this result is promising because other parasites increase mortality in anurans during overwintering (*Batrachochytrium dendrobatidis*; Rumschlag & Boone, 2018; Wetsch et al., 2022) and trematodes can reduce overwinter survival in some fish species (Lemly & Esch, 1984). Aside from their positive effects on mass at metamorphosis, which increased behavioral performance in the terrestrial environment, our results suggest that these natural enemies (i.e., predators and parasites) have minimal carryover effects on amphibians after metamorphosis.

### 4.3 Greatest impacts of parasites or predation on population growth may be through effects on age to maturity

Predators and parasites can regulate population growth through effects on survival and indeed, there are many instances where wildlife populations have been dramatically reduced because of natural enemies (Pettorelli et al., 2011; Tompkins et al., 2002). In the present study, we observed decreases in northern leopard frog survival to metamorphosis with bluegill exposure and in some parasite present communities; however, these effects had a limited influence on population growth in this species. Likewise, exposure to parasites and predators in the larval environment did not affect overwinter survival, suggesting no impacts on population growth beyond their impacts on tadpole survival. Yet, the indirect positive effects of predators and parasites on northern leopard frog body condition may have substantial effects on population growth by increasing their body size, which can reduce the time to sexual maturity (and increase fecundity, which we did not model in the present study). Reducing the minimum time for leopard frogs to reach reproductive maturity from 2 years to 1 year had a much larger effect on population growth than did the direct effects of bluegill and parasites on survival to metamorphosis.

Age to sexual maturity has long been considered a crucial species characteristic for determining population growth (Cole, 1954), especially for r‐selected species, such as amphibians, with early maturity and high reproductive rates (Oli & Dobson, 1999; Stahl & Oli, 2006). Our elasticity analysis revealed that factors that influence pre‐juvenile survival (i.e., the combined probability of embryo survival, tadpole survival, and metamorph survival) contribute the most to population growth (i.e., λ). However, factors that influence time to reproductive maturity influence multiple matrix elements (i.e., probability of remaining a juvenile, probability of becoming an adult, and juvenile fecundity). When combined, the influence of these three matrix elements on λ is larger than the influence of pre‐juvenile survival alone (elasticity values: 0.430 > 0.351). Our results suggest that the sublethal effects of predators and parasites on host/prey populations may compensate for direct negative effects of natural enemies on population dynamics leading to unanticipated population growth and stability in a host–parasite system.

## 5 CONCLUSIONS

Many ecological experiments with parasites do not mimic natural environmental conditions. To eliminate the misconception that parasites have blanket effects across all populations, we need to explore the environmental context where hosts and parasites exist, which is possible in manipulative field experiments. To understand how changing food web structure influences population and community dynamics, experiments are needed that incorporate natural complexity of ecological communities and measure multiple endpoints across life stages. This study is the first in this system to evaluate the influence of these parasites across life stages and to link the sublethal impacts of parasites and predators on amphibian population dynamics. If this experiment had ended at metamorphosis, one might conclude that changing community composition through predator and parasite additions has negative consequences for anuran populations through direct effects on survival; however, by indirectly influencing anuran body condition, changing community composition may have long‐term benefits that foster their success later in life. Without long‐term and experiments that incorporate environmental complexity, we may make inaccurate conclusions about the state of our ecological communities, which is problematic given the rapid changes occurring in ecosystems across the globe.

## AUTHOR CONTRIBUTIONS

**Miranda Strasburg:** Conceptualization (equal); data curation (lead); formal analysis (lead); funding acquisition (equal); investigation (lead); methodology (lead); project administration (equal); resources (equal); supervision (equal); visualization (lead); writing – original draft (lead); writing – review and editing (equal). **Michelle D. Boone:** Conceptualization (equal); funding acquisition (equal); project administration (supporting); resources (equal); supervision (equal); writing – review and editing (equal).

## CONFLICT OF INTEREST

Authors have no conflict of interest to declare.

## Supporting information


Figure S1

## Data Availability

Data are deposited in the Dryad Digital Repository (https://doi.org/10.5061/dryad.2280gb5vt).
