# Supplementary material for: Can predators stabilize host–parasite interactions? Changes in aquatic predator identity alter amphibian responses and parasite abundance across life stages
Source: Ecol Evol. 2022 Nov 15;12(11):e9512. doi: 10.1002/ece3.9512 (PMC9666717; doi:10.1002/ece3.9512)

Supplemental Figure 1. Influence of larval treatment on survival and change in mass through overwintering. A) Proportion of northern leopard frog tadpoles exposed to predator treatments (none, bluegill, crayfish, or mosquitofish) and parasite treatments (absent, present) that survived overwintering. B) Average change in mass of northern leopard frogs exposed to predator and parasite treatments from metamorphosis through overwintering. Plotted values are means ± 1 S.E.

Supplemental Figure 1.

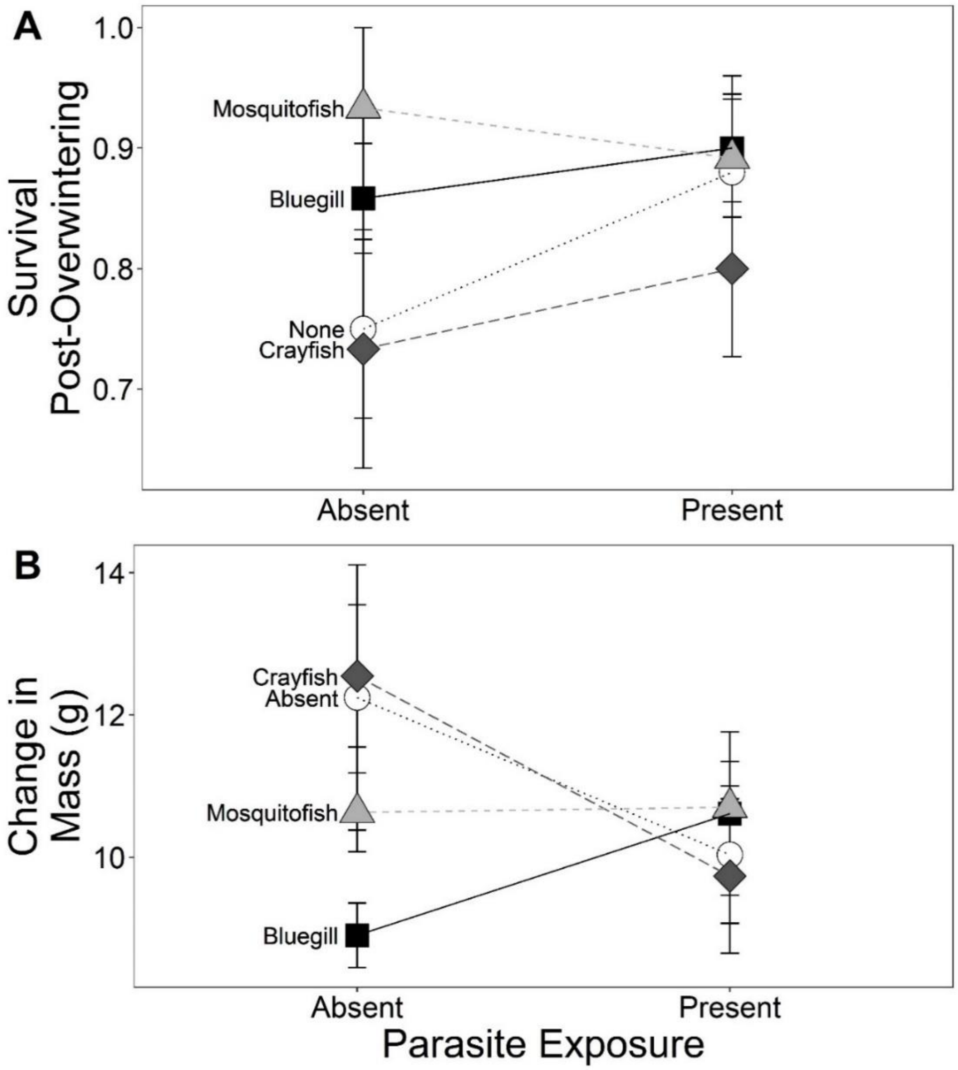

Supplement: Supplementary file 1 — Figure S1 [file ECE3-12-e9512-s001.pdf]
